# Predicting comorbid mental health difficulties in people with autoimmune arthritis

**DOI:** 10.1007/s00296-023-05519-8

**Published:** 2024-01-18

**Authors:** Caitlin A Hibbs

**Affiliations:** Heriot-Watt University, Edinburgh, UK

**Keywords:** Autoimmune disease, Arthritis, Age of onset, Mental health, Adverse childhood experiences

## Abstract

Little is known about variables impacting the association between mental health difficulties and autoimmune conditions. This study investigates whether, age of onset, adverse childhood experiences (ACEs), and ‘illness invisibility’ predict comorbid mental health difficulties in people with autoimmune arthritis. Participants with autoimmune arthritis (*N* = 209) were recruited via social media platforms. Age of onset of arthritis and the temporal order of mental health difficulties (if applicable) were collected alongside a measure of personality and ACEs. A novel measure of illness invisibility was developed for this study. A cross-sectional mixed-subject design was utilised. 53.5% of the sample endorsed lifetime mental health difficulties. Logistic regression analyses revealed participants with a younger age of onset of arthritis had significantly higher odds of developing a mental health problem (OR 0.93, 95% CI 0.90–0.96). Independently, Illness Invisibility, endorsed by 89.9% of participants, significantly predicted postmorbid mental health difficulties (OR 1.08, 95% CI 1.01–1.19). Adverse Childhood Experiences were frequently endorsed within the sample with 37.8% reporting ≥ 3 cumulative ACEs. Every unit increase in ACEs increased the odds of having comorbid mental health difficulties (OR 1.27, 95% CI 1.09–1.47). Young people who are diagnosed with autoimmune arthritis maybe more likely to experience subsequent mental health difficulties. The ‘invisibility’ of their illness and exposure to ACEs also is associated with their risk for mental health complications. These findings highlight the importance of mental health screening for young people being investigated for arthritis and interdisciplinary care, especially for young people.

**Supplementary Information:**

The online version contains supplementary material available at 10.1007/s00296-023-05519-8.

## Introduction

Several studies indicate a relationship between autoimmune diseases, including arthritis, and mental health disorders, with an overwhelming consensus for a negative impact [1, 2]. Slightly less is known about variables that impact the relationship between autoimmune arthritis (AA) and mental health (MH) difficulties.

Autoimmune arthritis is widely associated with comorbid mood and anxiety disorders [3, 4] poor quality of life and impaired health related quality of life [5, 6]. Chronic pain and the debilitating nature of the disease (e.g., reduced physical functioning) are thought to contribute to the causal pathway from arthritis to MH difficulties [7, 8]. A longitudinal study reported a cumulative risk of self-reported depression of almost 40% following 9 years of follow-up in those with Rheumatoid Arthritis [8]. Pain and fatigue intensity were the strongest predictors of self-reported depression.

Arthritis has also been associated with suicidal behaviours [9, 10]. Fuller-Thompson et al. [10] following controlling for demographics and MH variables (e.g., ACEs, depression, anxiety, and pain) discovered significantly higher odds of suicide attempts in those with arthritis compared to healthy controls. Within-subject comparisons revealed ACEs, mental illness, and current chronic pain increased the odds of suicide attempts in those with arthritis. Moreover, for every decade in age of the participant, there was a decrease in the odds of suicide attempt by 19%, highlighting the vulnerability of the young.

Studies in psychoneuroimmunology suggest a causal pathway from early trauma to autoimmune disease [11] via changes in cytokine release [12] and/or greater T-cell lymphocytes and lower cortisol levels [13]. Adverse Childhood Experiences (ACEs) [14] are negative life events during the first 18 years of life (e.g. physical, emotional, and sexual, abuse; emotional and physical neglect; exposure to domestic violence). Physical and mental ill-health are associated with ACEs in both childhood and adulthood [15, 16]. Baiden et al. [17] investigated the relationships between childhood abuse and arthritis. Using log-binomial regression, the authors found a 36% (physical abuse) and 60% (sexual abuse) increase in risk for arthritis compared to controls.

Illness Invisibility (II), an absence of recognition or awareness of the illness within family, social and medical contexts, has been identified as a source of less obvious challenges [18]. Disclosure or concealment of an ‘invisible’ condition may lead to invalidation, stigma and stress. Invisible illnesses allow a person to present as normal (concealment) which may be desired, particularly among young people, where being part of a peer group is so important. ‘Passing as normal’ can create problems, however, when concealment is no longer possible, e.g. when taking sick days. Given the person has not presented as having an illness, disclosure may be met with incredulity and further invalidation. All of these outcomes are likely to create additional stress for the individual. Sloan et al. [19], in an ethnographic study of individuals living with Lupus, identified a principal theme of invalidation when discussing medical interactions with family and friends. Participants were often told “but you don’t look sick” which resulted in interpersonal difficulties. Illness invisibility may be particularly challenging to deal with for children and adolescents diagnosed with AA. Given the societal association with older age, children and adolescents may be more likely to experience doctors, family members and peers disbelieving their symptoms or diagnosis. Also, as coping strategies are still in development, young people may have fewer capacities to mitigate the impact of invalidation and stigma. Due to the relative novelty of the concept of II, there are few studies and no established measures, making this an important avenue of future research.

This study investigates potential variables relevant to the comorbidity of AA and MH difficulties, specifically: age at onset, ACEs and II. The study begins by investigating whether greater childhood adversity, measured using a standard ACEs questionnaire predicts the comorbidity of AA and MH difficulties before examining whether a younger age of onset of AA predicts a greater likelihood of subsequent MH difficulties. Using a measure uniquely designed for the purpose of this study, II was hypothesised to significantly predict the occurrence of subsequent MH difficulties in those diagnosed with AA. Personality has a long-standing connection to MH difficulties [20]. Neuroticism and conscientiousness display the strongest relationships with physical health outcomes [21], whether this is via biological mechanisms as mentioned earlier or as a result of increased compliance to treatment regimens is unclear. To prevent any possible confounds, personality was controlled for in all analyses.

## Methods

### Participants

Inclusion criteria for the study were individuals 18 years old and above with a self-reported physician diagnosis of an autoimmune arthritis condition. 268 individuals consented to participate in the study, of which 209 completed the final page of the questionnaires giving a completion rate, in accordance with the CHERRIES guidelines, of 77.99% (please see supplementary material for a completed CHERRIES table). 59 participants did not satisfy inclusion criteria due to ineligibility (i.e., neither 18 years old nor a physician diagnosis of AA; *N* = 9); incomplete data (*N* = 47) or not having an AA diagnosis (e.g., systemic lupus; *N* = 1). An additional two participants were removed due to inconsistent data, i.e., likely due to misreading certain questions. Five participants did not provide demographic information and whether the concept ‘illness invisibility’ “resonated” with them but otherwise had complete data. These data were included as the II resonance item is not included within the total score for the measure.

### Main outcome variable

The primary outcome variable was whether or not the person had experienced MH difficulties and whether those difficulties pre-dated (premorbid) or post-dated (post-morbid) their diagnosis of arthritis. To establish this, participants were asked about the onset of any MH difficulties, and, if so, whether they had received a diagnosis or treatment and at what age their difficulties had occurred. Depending on participant responses, they were classified into three groups (pre-morbid MH difficulties (*N* = 65), post-morbid MH difficulties (*N* = 47), and no MH difficulties (*N* = 97). To maintain a conservative test of hypotheses, individuals who stated they had experienced MH difficulties but who had not received either a medical diagnosis, medication, or treatment (*N* = 17) were included in the no MH condition.

### Study factors

Information was collected on the following demographic variables: age, gender, ethnicity, level of education. Further details on their arthritis diagnosis, age of onset, age of diagnosis was also collected. Participants then completed measures of personality, II and ACEs. There were a total of 50 questions. No randomisation of items or questionnaires was used. All information was collected online via Qualtrics.

### Measures

#### Adverse Childhood Experiences

Adverse Childhood Experiences were examined using the 10-item NSCH-ACEs (http://www.nschdata.org) questionnaire [22] and includes questions on whether the individual experienced: sexual, physical, and psychological, abuse; physical and emotional neglect; exposure to parental, suicide, substance abuse, domestic abuse, depression, and incarceration; household dysfunction. The maximum score possible was 10.

#### Illness Invisibility

An II questionnaire was developed based on data from previous studies considering the invisible nature of some chronic conditions [18, 19] and the personal experience of the author. The questionnaire comprised a total of ten questions: participants were asked whether the concept of ‘illness invisibility’ resonated with them, whether they had been misdiagnosed prior to their arthritis diagnosis, and whether they had ever been told “but you don’t look sick”. Six further statements followed, covering themes of invalidation, stigmatisation, inter- and intra-personal challenges in different societal settings (e.g., personal, medical, and work), also rated on a five-point Likert scale. Illness Invisibility was scored by totalling scores from the six statements combined with the score for how often participants had been told, “but you don’t look sick” (a score of zero was given if they responded no to this; see supplementary material). The maximum possible score was 34.

#### Personality

Personality traits of extraversion, conscientiousness, neuroticism, agreeableness, and openness were measured using the Big Five Inventory (BFI-10) [23]. The BF1-10 comprises 10-items measured on a 5-point Likert scale. Test–retest reliability of this measure has a mean of 0.75 following 6–8 weeks.

### Procedures

Participants were opportunistically and snowball sampled via several social media platforms, namely: Facebook, LinkedIn, Instagram, and Twitter. The majority of participation (*N* = 187) followed recruitment via four Facebook support groups: ‘Autoimmune Disease Support Group’; ‘Arthritis Foodie Forum’; ‘Rheumatoid Arthritis in the UK’; ‘Arthritis and Joint Disease’; ‘Psoriatic Arthritis Community: support, resources and discussion’.

Participants interested in taking part in the study were first presented with the information sheet informing them of the procedure and nature of the study, including information about its sensitive nature. Participants had the opportunity to give consent at this point and were provided with a personalised PIN enabling them to withdraw their data. If inclusion criteria were not met, participants were informed that unfortunately they were ineligible and were redirected to the end of the survey. Participants were fully debriefed at the end of the survey. Data were collected between December 2021 and January 2022.

### Ethics

Ethical approval was received from the School of Social Sciences Ethics Committee at Heriot Watt University. The author chose to disclose her personal experiences in the advertisement hoping this would mitigate potential stigma participants may feel about disclosing MH conditions. To account for potential sampling bias (i.e., increased participation from individuals with comorbid MH difficulties), a sentence highlighting comorbidity was not an inclusion criterion was also included. As 97 of the sample did not have MH conditions, the sampling method appeared successful in this regard. The sensitive nature of the study was also highlighted, and participants were advised not to participate if they thought this may cause any distress.

### Data analysis

A logistic regression was used to determine whether ACEs score significantly predicted comorbid MH difficulties. Personality traits were included as covariates. All participants (*N* = 209) were included in the analysis. To test predictors of postmorbid MH difficulties (i.e. where age of onset and/or II predicted the occurrence of subsequent MH difficulties), logistic regression analyses were performed (those within the premorbid MH condition were excluded from these analyses). Personality traits were entered as covariates. Data was analysed using IBM SPSS-28.0.

## Results

Participants in the analytic sample (*N* = 209) were diagnosed with one of four different types of arthritis (72.7% Rheumatoid Arthritis, RA; 15.8% Psoriatic Arthritis, PsA; 5.7% Juvenile Idiopathic Arthritis, JIA; 5.7% of participants had other diagnoses of arthritis. (e.g., ankylosing spondylitis, seronegative inflammatory arthritis). The sample comprised 94.7% females (*n* = 198) with an overall mean age of 41.69 years (SD = 12.46 years; Table 1).

**Table 1:** Descriptive statistics of full sample and sub-samples

	No mental health (*n* = 97)			Mental health (*n* = 112)									Full sample (*n* = 209)		
				Pre-morbid MH (*n* = 65)			Post-morbid MH (*n* = 47)			Total (*n* = 112)					
	*M*	SD	Range	*M*	SD	Range	*M*	SD	Range	*M*	SD	Range	*M*	SD	Range
Age^a^	44.62	39.15	51.00	39.65	12.67	47.00	38.47	13.54	49.00	39.15	12.99	49.00	41.69	12.46	53.00
Age of onset^a^	33.05	11.68	51.00	30.74	11.91	52.00	19.51	14.96	59.00	26.03	14.34	59.00	29.29	13.60	60.00
Age of diagnosis^a^	36.42	10.91	48.00	34.05	11.15	45.00	25.40	16.10	63.00	30.42	14.06	63.00	33.21	13.01	63.00
Diagnosis time^a^	3.37	4.91	21.00	3.31	5.25	29.00	5.89	7.68	33.00	4.39	6.48	33.00	3.92	5.81	33.00
Age of MH onset^a^	N/A			19.77	9.88	50.00	26.11	14.77	58	22.43	12.51	59.0		N/A	
Extraversion	5.53	2.21	8.00	4.60	2.13	8.00	5.49	2.20	8.00	4.97	2.19	8.00	5.23	2.11	8.00
Neuroticism	5.16	1.98	8.00	6.69	2.16	8.00	6.53	2.10	8.00	6.63	2.14	8.00	5.95	2.18	8.00
Conscientiousness	7.32	1.43	4.00	6.22	1.52	7.00	6.94	1.87	7.00	6.52	1.70	7.00	6.89	1.63	7.00
Agreeableness	6.21	1.91	8.00	5.66	2.19	8.00	6.19	1.79	7.00	5.88	2.04	8.00	6.03	1.98	8.00
Openness	5.48	2.0	8.00	5.89	1.73	8.00	6.09	1.80	7.00	5.97	1.76	8.00	5.75	1.89	8.00
Illness Invisibility Score	23.98	6.33	28.00	25.88	4.67	21.00	27.11	5.00	18.00	26.39	4.83	22.00	25.27	5.69	28.00
Adverse Childhood Experiences Score	1.68	1.97	10.00	3.43	2.50	8.00	2.17	2.19	9.00	2.90	2.45	9.00	2.33	2.31	10.00

^a^In years

### Adverse childhood experiences

Adverse Childhood Experiences were frequently endorsed within the sample, with 37.8% reporting cumulative ACEs. Household dysfunction (namely divorce and exposure to a depressed or suicidal parent), emotional neglect and emotional abuse were the most prevalent, respectively. Those with comorbid MH difficulties exhibited a significantly greater prevalence across all ACEs, with those within the premorbid MH condition observationally displaying the highest prevalence of all (Table 2).

**Table 2:** Prevalence of Adverse Childhood Experiences

	No mental health (*n* = 97)		Mental health (*n* = 112)						Full sample (*n* = 209)	
			Pre-morbid MH (*n* = 65)		Post-morbid MH (*n* = 47)		Total (*n* = 112)			
	*n*	%	*n*	%	*n*	%	*n*	%	*n*	%
Did a parent of other adult in the household **often**…^a^ Swear at you, insult you, put you down, or humiliate you? **Or** Act in a way that made you afraid you might be physically hurt?	24	24.7	32	49.2	13	27.7	45	40.2	69	33.0
Did a parent of other adult in the household **often**…^a^ Push, grab, slap, or throw something at you? **Or** **Ever** hit you so hard that you had marks or were injured?	22	22.7	24	36.9	8	17.0	32	28.6	54	25.8
Did an adult or person at least 5 years older than you **ever**…^a^	11	11.3	14	21.5	5	10.6	19	17.0	30	14.4
Touch or fondle you or have you touch their body in a sexual way? **Or** Try to or actually have oral, anal, or vaginal sex with you?										
Did you **often** feel that…^a^ No one in your family loved you or thought you were important or special? **Or** Your family didn’t look out for each other, feel close to each other, or support each other?	17	17.5	37	56.9	17	36.2	54	48.2	71	34.0
Did you **often** feel that…^a^ You didn’t have enough to eat, had to wear dirty clothes, and had no one to protect you? **Or** Your parents were too drunk or high to take care of you or to take you to the doctor if you needed it?	4	4.1	15	23.1	4	8.5	19	17.0	23	11.0
Were your parents ever separated or divorced?^a^	31	32.0	34	52.3	15	31.9	49	43.8	80	38.3
Was your mother or stepmother^a^: **Often** pushed, grabbed, slapped, or had something thrown at her? **Or** **Sometimes or often** kicked, bitten, hit with a fist, or hit with something hard? **Or** **Ever** repeatedly hit over at least a few minutes or threatened with a gun or knife?	8	8.2	9	13.8	3	6.4	12	10.7	20	9.6
Did you live with anyone who was a problem drinker or alcoholic or who used street drugs?^a^	19	19.6	23	35.4	14	29.8	37	33.0	56	26.8
Was a household member depressed or mentally ill or did a household member attempt suicide?^a^	25	25.8	29	44.6	20	42.6	49	43.8	74	35.4
Did a household member go to prison?^a^	2	2.1	6	9.2	3	6.4	9	8.0	11	5.3
0^b^	34	35.1	7	10.8	13	27.7	20	17.9	54	25.8
1^b^	23	23.7	13	20.0	11	23.4	24	21.4	47	22.5
2^b^	14	14.4	9	13.8	6	12.8	15	13.4	29	13.9
$\ge 3$^b^	26	26.8	36	55.4	17	36.2	53	47.3	79	37.8

^a^*n* = those who responded ‘yes’

^b^*n* = cumulative ACEs

Adverse Childhood Experiences significantly predicted comorbid MH problems (omnibus Chi-square = 47.07, *df* = 6, *p* < 0.001) and the model accounted for between 20.2 and 26.9% of the variance. Overall, 68.4% of cases were correctly predicted by the model, with 72.3% and 63.9% of those with and without MH difficulties accurately predicted, respectively. Adverse Childhood Experiences score (Wald = 9.60, *df* = 1, *p* = 0.002), conscientiousness (Wald = 8.24, *df* = 1, *p* = 0.004) and neuroticism (Wald = 14.29, *df* = 1, *p* < 0.001) were significant predictors of MH difficulties in those with AA. Every unit increase in ACEs was associated with an increase in the odds of having MH difficulties by 27% (OR 1.27, 95% CI 1.09–1.47; Table 3).

**Table 3:** Coefficients for the model analysing whether ACEs predict comorbid mental health difficulties in individuals with autoimmune arthritis

Variable	*b*	Wald	*p*	Exp (*b*)	95% CI	
					Lower	Upper
Adverse childhood experiences	0.24	9.60	0.002	1.27	1.09	1.47
Neuroticism	0.30	14.29	< 0.001	1.35	1.16	1.58
Extraversion	0.04	0.31	0.577	1.04	0.90	1.21
Conscientiousness	− 0.30	8.24	0.004	0.74	0.60	0.91
Agreeableness	0.03	0.16	0.689	1.04	0.88	1.22
Openness	1.33	2.44	0.118	1.14	0.97	1.35

*R*^2^ = 0.202 (Cox & Snell), 0.269 (Nagelkerke)

### Age of onset of arthritis

The mean age of onset for the entire sample was 29.29 years (SD = 12.46). The post-morbid MH group had the lowest age of onset at 19.51 years (SD = 14.96 years), with those without MH difficulties showing the highest age at onset of 33.05 years (SD = 11.68 years; Table 1). One-hundred-and-forty-four cases were analysed, and the model significantly predicted the temporal onset of MH difficulties (omnibus Chi-square = 42.52, *df* = 6, *p* < 0.001). The model accounted for 25.6–35.6% of the variance, with 55.3% of those with subsequent MH difficulties correctly predicted. For those without any MH difficulties, 88.7% of cases were correctly predicted. Overall, 77.8% of predictions were accurate. Only age of onset (Wald = 17.70, *df* = 1, *p* < 0.001) and neuroticism (Wald = 7.34, *df* = 1, *p* = 0.007) significantly predicted MH outcomes in individuals with AA (Table 4). Every 1-year increase at age of onset was associated with a decrease in the odds of developing MH difficulties by 7% (OR 0.93, 95% CI 0.90–0.96). Additionally, each unit increase of neuroticism was associated with an increase in the odds of developing MH difficulties by 37% (OR 1.37, 95% CI 1.07–1.68).

**Table 4:** Coefficients of the model predicting whether age of onset predicts postmorbid mental health difficulties in individuals with autoimmune arthritis

Variable	*b*	Wald	*p*	Exp (*b*)	95% CI	
					Lower	Upper
Age of onset	− 0.07	17.70	< 0.001	0.93	0.90	0.96
Neuroticism	0.31	7.34	.007	1.37	1.07	1.68
Extraversion	0.07	0.44	0.52	1.07	0.88	1.31
Conscientiousness	− 0.04	0.09	0.76	0.96	0.73	1.26
Agreeableness	− 0.03	0.07	0.80	0.97	0.77	1.22
Openness	0.20	2.62	0.11	1.22	0.96	1.54

*R*^2^ = 0.256 (Cox & Snell), 0.356 (Nagelkerke)

### Illness Invisibility

89.9% of the participants stated that II resonated with them. Moreover, 82.8% of participants disclosed that they had been told “but you don’t look sick” either by a friend, family member or colleague. Illness Invisibility scores tended to be very high across all groups with a mean of 25.27 (SD = 5.69). Those with comorbid MH problems scored significantly higher on II on average compared to those without MH difficulties (*M* = 26.39, SD = 4.83, *M* = 23.98, SD = 6.33, respectively), with individuals with postmorbid MH scoring the highest overall (*M* = 27.11, SD = 5.0; Table 1).

To establish whether II predicted postmorbid MH difficulties, a logistic regression analysis was completed (*n* = 144). Personality traits were entered as covariates. Illness invisibility predicted post-morbid MH difficulties in participants with premorbid AA diagnosis (omnibus Chi-square = 24.73, *df* = 6, *p* < 0.001). Between 15.8 and 22% of the variance was accounted for by the model. Illness Invisibility (Wald = 4.28, *df* = 1, *p* = 0.039) and neuroticism (Wald = 10.03, *df* = 1, *p* = 0.002) were the only significant predictors. Each unit increase in II (OR 1.08, 95% CI 1.01–1.19) and neuroticism (OR 1.41, 95% CI 1.14–1.75) resulted in an increase in the odds of having MH difficulties (Table 5). The model only correctly predicted 48.9% of those with mental health difficulties, however. The prediction for those without MH difficulties was 89.7% accurate, with an overall percentage of 76.4%.

**Table 5:** Coefficients of the model predicting whether illness invisibility predicts postmorbid mental health difficulties in individuals with autoimmune arthritis

Variable	*b*	Wald	*p*	Exp (*b*)	95% CI	
					Lower	Upper
Illness invisibility Score	0.08	4.28	0.039	1.08	1.004	1.17
Neuroticism	0.35	10.03	0.002	1.41	1.14	1.75
Extraversion	0.12	1.45	0.23	1.12	0.93	1.35
Conscientiousness	− 0.12	0.86	0.35	0.89	0.70	1.14
Agreeableness	0.04	0.15	0.70	1.05	0.84	1.31
Openness	0.163	2.14	0.14	1.18	0.95	1.46

*R*^2^ = 0.158 (Cox & Snell), 0.220 (Nagelkerke)

Given that II was a significant predictor of post-morbid MH difficulties, a post hoc analysis including II, age of onset and personality traits as covariates was run. Neuroticism (OR 1.34, 95% CI 1.07–1.68) and age of onset (OR 0.93, 95% CI 0.90–0.97) remained significant. Whilst II was not significant in this model (Table 6), the revised overall model was stronger (omnibus Chi-square = 43.88, *df* = 7, *p* < 0.001) with an overall accurate prediction of 79.9% of cases, with 61.7% and 88.7% of those with and without MH difficulties predicted correctly, respectively. Furthermore, 26.3–36.6% of the variance was accounted for by the model.

**Table 6:** Coefficients of the revised model analysing predictors of postmorbid mental health difficulties in individuals with autoimmune arthritis

Variable	*b*	Wald	*p*	Exp (*b*)	95% CI	
					Lower	Upper
Age of onset	− 0.07	15.33	< 0.001	0.93	0.90	0.97
Neuroticism	0.29	6.27	0.012	1.34	1.07	1.68
Illness Invisibility Score	0.05	1.12	0.293	1.05	0.96	1.15
Extraversion	0.09	0.72	0.396	1.09	0.89	1.35
Conscientiousness	− 0.05	0.13	0.719	0.95	0.73	1.25
Agreeableness	− 0.01	0.01	0.939	0.99	0.78	1.25
Openness	0.18	2.13	0.144	1.20	0.94	1.53

*R*^2^ = 0.262 (Cox & Snell), 0.365 (Nagelkerke)

## Discussion

This study investigated the impact of potential predictors (i.e., ACEs, age of onset and II) of experiencing subsequent and comorbid MH difficulties, in people with AA. ACEs significantly predicted comorbid MH difficulties; neuroticism, as well as conscientiousness, were also significant predictors in this model. Age of onset of diagnosed AA was a significant predictor of the development of subsequent MH difficulties, specifically, those with a younger age of onset had increased odds of developing MH difficulties. Illness Invisibility, a novel phenomenon, was commonly experienced and significantly and independently predicted MH outcomes. When included in a model with age of onset, II ceased to remain significant, however, it contributed to a stronger model which was more successful at predicting individuals reporting postmorbid MH difficulties. Furthermore, neuroticism consistently and significantly predicted postmorbid MH difficulties in both models.

Within the current study, greater than 50% and 35% of participants reported ≥ 2 and ≥ 3 ACEs, respectively. These rates are higher than those found in a national study of ACEs [11] as risk factors for autoimmune disease. Given the higher occurrence of ACEs within our solely AA sample, our findings may lend support to the hypothesis that ACEs may be a causal factor for AA. Moreover, those within the premorbid MH sample displayed the greatest prevalence of ACEs, arguably fitting with psychoneuroimmunology assumptions that both ACEs and MH disorders may increase risk of ADs (e.g. [1, 2]).

Autoimmune disorders have previously been associated with an increased risk of depression [2, 24], anxiety disorders [24, 25] and bipolar disorder [24]. While the prevalence of MH disorders in these studies ranged from a third to 45%, in our sample, 27.8% of individuals with AA reported a medical diagnosis for a mental disorder. Furthermore, more than half of the sample reported having received a psychiatric diagnosis or treatment for their MH difficulties at some point in their lifetime. Given self-declaration of previous treatment(s) was used, and individuals without a diagnosis were not excluded, directly comparing previous prevalence findings is difficult. Notwithstanding this limitation, our findings are comparable with other studies.

Uniquely, an earlier age of onset, as well as higher levels of neuroticism, resulted in a greater likelihood of postmorbid MH problems in individuals with AA in this sample. There are several possible explanations for this. Firstly, in this sample, participants with an earlier age of onset had been living with AA longer. Living longer with a debilitating chronic condition may increase the likelihood of experiencing MH difficulties because of increased exposure to pain and inflammation, restrictions on typical activities and persistent invalidation [5, 7, 8, 26]. Second, children and adolescents with AA exhibit significantly reduced Health-Related Quality of Life (HRQOL) [27]. Those with rheumatologic conditions (e.g., JIA) are particularly adversely affected. Lower HRQOL is independently associated with psychopathology in JIA [28, 29] as are disease severity and activity levels [30]. Whilst the mean age in our post-morbid mental health group was 19.51 years, given the common delay between symptom onset and diagnosis, participants in this group were likely to have started with symptoms during childhood or adolescence. Third, the onset of a major health condition in adolescence might be particularly challenging due to other biological and cognitive changes occurring during this time and thus may increase the odds of subsequent MH difficulties [31]. Coping strategies are still developing, and young people may tend to utilise ineffective (e.g., rumination) rather than effective (e.g. positive self-talk) coping strategies due to a lack of awareness and or development [32]. Greater pain also appears to limit the use of coping mechanisms, or equally, certain coping mechanisms may be ineffective in coping with pain which perpetuates the pain [33].

Illness Invisibility was a new measure designed for this study based on previous qualitative research [18, 19] and when considered alone statistically predicted subsequent MH difficulties in individuals with AA. Caution is required in interpreting this finding as the measure has not been independently validated. Illness Invisibility could be associated with onset of mental health difficulties for several reasons. Firstly invalidation—specifically, discounting and a lack of understanding—are associated with and predict significantly poorer mental health among individuals with rheumatic diseases (e.g., RA, PsA, ankylosing spondylitis) [34]. Secondly, stigma has also been identified as a social phenomenon with relatively high levels in individuals living with RA [35] and is associated with discrimination in several areas (e.g., employment, education, and interpersonal relationships) in those with chronic illness [36, 37].

Relatedly, individuals with JIA, who by definition have early onset AA, may ‘strive for normality’, often hiding their physical pain and illness from people around them in order to avoid being perceived or treated differently [38]. Striving for normality may encourage maladaptive coping strategies (e.g., avoidance of support seeking, ignoring pain, and over exertion) and promote self-invalidation (e.g. by resisting accepting/acknowledging being different) [39].

Illness Invisibility ceased to significantly predict MH difficulties when included with age of onset in the combined model. This could be an artefact of the unreliability of the measure, however, there may be other interpretations of this: first, II may be fully accounted for by age of onset, and thus may have no direct effect on postmorbid MH; second, there is an interaction between age of onset and II scores. Given the overall model was stronger with II, arguably, the latter explanation is more plausible, where age of onset masks the impact of II. Illness invisibility may be more prominent in younger people with AA due to the misconception that arthritis is an ‘old person's disease’ and the inherent lack of awareness in lay people (and sometimes health professionals) that AA does not discriminate by age [40].

In line with previous research [20], neuroticism increased the odds of reporting MH difficulties. It is outside of the scope of the current study to determine whether neuroticism scores are a by-product of MH disorders or whether both are rooted in complex early life trauma (e.g. ACEs) [41, 42]. Conscientiousness acted as a protective factor against comorbid MH difficulties in those with AA. Conscientious individuals may be inclined to adhere to psychological guidance in the face of adversity, which may lead to healthful behaviour patterns, which in turn may act as a protective factor against MH difficulties [43, 44].

## Limitations

The most significant limitations of the study are first the cross-sectional design meaning causality cannot be inferred—we cannot absolutely determine that a younger age of onset of AA caused postmorbid MH difficulties or that ACEs caused comorbid MH and AA. Secondly, whilst II was well recognised within the sample the questionnaire itself was novel and not independently validated. Thirdly, as we did not have a comparison group, our findings may not be unique to AA and may simply reflect the challenges of living with any chronic condition.

With respect to the sample, the majority of participants were recruited via support groups, and it may be a reasonable assumption that individuals seeking support may be inclined to be already struggling with living with AA. Data may, therefore, be skewed to reflect a greater proportion of individuals with MH difficulties and not be representative of the wider population of those with AA. Additionally, diagnosis was self-report, and we cannot rule out the possibility that some participants did not have an autoimmune arthritis as we did not cross check with physician records. Furthermore, the sample was almost entirely white and well-educated. Although AA is a universal disease, we cannot assume from the data that the variables tested here have the same effect on the relationship between AA and MH for other ethnicities, cultures, and lower socio-economic groups.

The sample was predominantly female, limiting the applicability of findings to other genders, however, autoimmune diseases are more common in women [45, 46].

## Conclusion

This study suggests that MH disorder diagnoses, and more broadly MH difficulties, are a common occurrence in individuals with AA, thus, interdisciplinary care between physical and MH services is imperative for these individuals. Our findings, supported by others (e.g. [47]) suggest that routine screening for MH difficulties, as part of physical health checks, alongside early intervention and targeted prevention, should be made a priority. As II resonates strongly with individuals with AA, psychoeducation immediately following diagnosis for patients and close family members may help them understand these less obvious challenges faced by individuals. Consecutively, this may reduce any potential invalidation (and self-invalidation), stigma and the potential odds of experiencing MH difficulties in these individuals with AA [48]. Particular attention needs to be paid to children and adolescents with AA as those with a younger age of onset had the highest II scores and may be more likely to subsequently report MH difficulties. Indeed, young people may benefit from interactions with other people their age with AA potentially decreasing feelings of abnormality and providing peer support. Finally, continued work to screen for and address ACEs early is crucial to attempt to prevent both MH difficulties and chronic illness [49].

### Supplementary Information

Below is the link to the electronic supplementary material.

Supplementary file1 (DOCX 15 KB)

Supplementary file2 (DOCX 23 KB)

## Data Availability

The data are not publicly available.
