# Supplementary material for: Predicting comorbid mental health difficulties in people with autoimmune arthritis
Source: Rheumatol Int. 2024 Jan 18;44(3):459–68. doi: 10.1007/s00296-023-05519-8 (PMC10866777; doi:10.1007/s00296-023-05519-8)
Supplement: Supplementary file 1 — Supplementary file1 (DOCX 15 KB) [file 296_2023_5519_MOESM1_ESM.docx]

# Supplementary Material: Illness Invisibility Questionnaire

1. Does the concept of ‘illness invisibility’ resonate with you? Yes / No
2. Were you ever misdiagnosed before your diagnosis of arthritis? Yes / No
3. Have you ever been told by a friend, family member or colleague, “but you don’t look sick”? Yes / No

3a. If yes, how often have you been told this?

- Not very often
- Sometimes
- Often
- Very often

1. How much do you agree with the following statements?

“I often felt like society, including family, friends and medical professionals, would gaslight my symptoms (i.e., make me doubt that they were even there)”

- Disagree strongly
- Disagree a little
- Neither agree nor disagree
- Agree a little
- Agree strongly

“I was often invalidated (i.e., dismissal or rejection of thoughts, feelings or behaviours) about my condition and symptoms by society, including family, friends and medical professionals”

- Disagree strongly
- Disagree a little
- Neither agree nor disagree
- Agree a little
- Agree strongly

“I have often felt blamed or punished in educational and / or work environments by those in authority for circumstances relating to my arthritis which were beyond my control (e.g. absences and lateness due to chronic pain and or fatigue etc.)”

- Disagree strongly
- Disagree a little
- Neither agree nor disagree
- Agree a little
- Agree strongly

“I often felt like those closest to me would forget what I was living with day to day”

- Disagree strongly
- Disagree a little
- Neither agree nor disagree
- Agree a little
- Agree strongly

“I often chose not to disclose my condition and try to ‘pass for normal’ due to potential stigmatisation and invalidation”

- Disagree strongly
- Disagree a little
- Neither agree nor disagree
- Agree a little
- Agree strongly

“The invisible nature of my condition often made me feel like people wouldn’t believe or understand me if told them”

- Disagree strongly
- Disagree a little
- Neither agree nor disagree
- Agree a little
- Agree strongly
